# Diseases of the Stomach and Intestines

**Published:** 1904-12-17

**Authors:** 


### DISEASES OF THE STOMACH AND INTESTINES.

(*Continued from page* 191.)

Alcohol.—In discussing the use of alcohol as a medicine, Monro[4] points out that various and contradictory opinions have been held as to the value of many drugs during the past century. As to alcohol, in the middle of the nineteenth century it was given in enormous doses as a stimulant in cases of fever; then a reaction set in, and to-day Liebig's theory of alcohol as a food is scarcely ever acted on, and is not indeed believed in the sense he originally propounded. It is still, however, given as a cardiac stimulant, but its action as a vaso-constrictor is transient and only due to reflex irritation of the alimentary mucous membrane. Its temporary action in constricting the vessels is followed by a prolonged fall in blood-pressure. Experimental evidence also clearly shows that alcohol, far from being a cardiac stimulant, is a direct poison to the heart muscle, producing dilatation and imperfect contraction. Its supposed action as a cardiac stimulant is due to its narcotic and sedative action, which may benefit a patient with high temperature and rapid pulse. It is not scientifically justified in cases of uncompensated valvular disease, and as it only raises blood-pressure reflexly it is not of much value in convalescence where the pulse is dicrotic or hyperdicrotic. Its action on the pulse-rate is insignificant, and its antipyretic action is secondary to its power of dilating the cutaneous vessels. In cold climates alcohol reduces the body temperature while in the tropics it predisposes to sunstroke, owing to cutaneous hyperæmia. Its main uses are to produce reflex stimulation in ordinary syncope; to induce sleep in certain cases; to produce cutaneous hyperæmia after exposure to severe cold, and to relieve the pain of neuralgia and dysmenorrhœa, but in the last-named conditions it is a highly dangerous remedy. Findlay[5] holds that it is of use in all conditions of arterio-capillary spasm as a vaso-dilator, but its only advantage over other drugs is that it is always handy. It increases the cerebral blood flow, but this is of doubtful advantage in cases of shock (vaso-motor exhaustion) because it is a direct poison to the cells of the nervous system. In the rigors of fever when the vessels are contracted it may bring relief to the patient, in the later stages when the vessels are fully dilated it is probably harmless as no further dilatation is possible. In arterio-sclerosis again its action is very slight, owing to the rigid condition of the tubes. Alcohol has no effect on the pulse-rate in health, but in functional disturbances it may retard the rate by its narcotising action. It has no effect on the tension either in health or disease. In high fever it only further embarrasses the heart by its action as a poison on the cardiac muscle. In toxic and septic conditions the action of alcohol is not definitely settled; it has been shown in animals that it destroys and prevents immunity, but the doses given were enormous, so that other factors—digestive disturbance, emaciation, etc.—cannot be eliminated. It almost certainly diminishes the alkalinity of the blood, and thus increases susceptibility to infection, and is thought by some to interfere with phagocytosis and the bacteriolytic properties of the blood. The question, however, is not finally settled, and at present alcohol is only known to be a narcotic, vaso-dilator, and reflex stimulant. The treatment of alcoholism (and also other drug habits) by atropine has been described by McBride,[6] who has obtained good results in many cases during the last 13 years. He cites four illustrative cases, one of which has remained cured since 1891. The treatment lasts about six weeks, and consists in giving atropine sulphate hypodermically in doses of $\frac{1}{200}$ gr. thrice daily, and increasing the dose during the first week to $\frac{1}{50}$ gr., or until the pupils are affected and the mouth becomes dry. It is continued at this stage during the remainder of the time. Strychnine nitrate is also injected, beginning at $\frac{1}{60}$ gr. and rising to $\frac{1}{20}$ gr., and fluid extract of cinchona is also given by the mouth in ordinary doses. Alcohol is not forbidden, but in a short time a nauseating distaste for it occurs and is permanent. The treatment requires the most careful personal supervision on the part of the physician, and is best carried out in institutions. Fenn[7] advocates a similar treatment, which he has tried for three and a half years. It consists of cheerful institution life, generous diet, baths of various kinds, and the injection of daturine sulphate grain $\frac{1}{200}$, or atropine grain $\frac{1}{300}$, and strychnine sulphate grain $\frac{1}{100}$, four times daily. A mixture containing sodium and gold chloride grain $\frac{1}{20}$, ammonium chloride grain i., aloin grain $\frac{1}{25}$, fluid extract of viburnum ♏ x., and tr. cinchonæ ♏ xl. After five days, if the patient continues to take alcohol, vomiting is induced by a little ipecacuanha. Moral and general hygienic treatment and attention to special symptoms are also necessary. Treatment lasts from four to eight weeks. In some cases failure results, but Australian statistics place the percentage of permanent cures at 80, and the author's experience points to about 60. In place of an institution the treatment may in some cases be carried out at home under a special resident administrator.

[4] Glasgow Med. Jour., May, 1904, p. 329. [5] Ibid., p. 335. [6] Brit. Med. Jour., April 30, 1904, p. 1006. [7] Ibid., p. 1008.

